# Social learning in humans and other animals

**DOI:** 10.3389/fnins.2014.00058

**Published:** 2014-03-31

**Authors:** Jean-François Gariépy, Karli K. Watson, Emily Du, Diana L. Xie, Joshua Erb, Dianna Amasino, Michael L. Platt

**Affiliations:** ^1^Department of Neurobiology, Center for Cognitive Neuroscience and Duke Institute for Brain Sciences, Duke UniversityDurham, NC, USA; ^2^Department of Biological Anthropology, Duke UniversityDurham, NC, USA

**Keywords:** social, dorsolateral prefrontal cortex, DLPFC, anterior cingulate cortex, anterior cingulate gyrus, temporoparietal junction, superior temporal sulcus, learning

## Abstract

Decisions made by individuals can be influenced by what others think and do. Social learning includes a wide array of behaviors such as imitation, observational learning of novel foraging techniques, peer or parental influences on individual preferences, as well as outright teaching. These processes are believed to underlie an important part of cultural variation among human populations and may also explain intraspecific variation in behavior between geographically distinct populations of animals. Recent neurobiological studies have begun to uncover the neural basis of social learning. Here we review experimental evidence from the past few decades showing that social learning is a widespread set of skills present in multiple animal species. In mammals, the temporoparietal junction, the dorsomedial, and dorsolateral prefrontal cortex, as well as the anterior cingulate gyrus, appear to play critical roles in social learning. Birds, fish, and insects also learn from others, but the underlying neural mechanisms remain poorly understood. We discuss the evolutionary implications of these findings and highlight the importance of emerging animal models that permit precise modification of neural circuit function for elucidating the neural basis of social learning.

## Introduction

The behavior of others provides a rich source of information that individuals can use to improve their behavior without direct experience. To illustrate, imagine for dinner you must choose between two restaurants that you have never tried before. Your friends tell you that one of them serves excellent food, but the other restaurant has unsanitary conditions. Without directly experiencing each outcome, most people can use this information to guide their decision about where to eat. This not only applies to learning food preferences, but also to mating decisions, fear learning, and problem-solving strategies (Olsson and Phelps, 2007; Gruber et al., 2009; Yorzinski and Platt, 2010; van den Bos et al., 2013; Wisdom et al., 2013). The process through which individuals learn from others rather than through direct experience is referred to as social learning. Social learning may underlie large-scale population phenomena such as variation in food preferences among geographically-distinct populations of animals and the diversity found in human cultures (Whiten, 2005; van de Waal et al., 2013). Many animal species learn from others, including chimpanzees, rats, monkeys, birds, and octopuses, suggesting that these abilities may have evolved as an adaptation to a range of different ecological niches (Fiorito and Scotto, 1992; Galef, 1995; Galef and Whiskin, 1995; Dally et al., 2008; Horner and de Waal, 2009; van Schaik and Burkart, 2011; Morgan et al., 2012; van de Waal et al., 2013). The adaptive advantage of social learning is also evident from the outcomes of game theory tournaments, in which algorithms that learn from opponents outperform those that do not (Rendell et al., 2010).

Several comprehensive reviews have been written on social learning and social cognition (Galef and Giraldeau, 2001; Whiten, 2005; Zentall, 2012; Stanley and Adolphs, 2013; van den Bos et al., 2013). Hence, our review focuses on studies that cover both the behavioral and neural mechanisms that mediate social learning. Here, we use “direct experience learning” to refer to any type of learning that individuals perform independently of others and “social learning” to refer to any form of learning influenced by other individuals.

## The neurobiology of learning from direct experience

The mechanisms by which individuals learn from direct experience have received a great deal of attention in recent years. Reinforcement learning models rely on updating a value representation of a given action when that action leads to favorable or unfavorable outcomes. These models use feedback from past outcomes to guide future decisions. Learning relies on the computation of a prediction error, which corresponds to the difference between an outcome and some previously-established expectation. The stored expectation is updated by this prediction error, multiplied by a learning rate that determines the speed at which outcomes can influence behaviors (Gläscher and Büchel, 2005; Pfeiffer et al., 2010; Funamizu et al., 2012). A variety of brain areas appear to be involved in reinforcement learning. This includes the striatum, which contains neurons that fire for specific sensory cues when they are paired with reward through conditioning (Aosaki et al., 1994). Dopamine neurons in the substantia nigra are known to encode prediction errors and are necessary for learning that requires prediction errors (Schultz et al., 1997; Schultz, 1998; Steinberg et al., 2013). In humans, functional magnetic resonance imaging experiments suggest that the activity of many other brain areas correlates with variables computed from learning theory including the amygdala (Gläscher and Büchel, 2005). Anterior cingulate cortex (ACC) lesions in monkeys impair the learning of task-switching paradigms, suggesting that the ACC might be important in monitoring errors and for attention in changing environments (Rushworth et al., 2003).

However, reinforcement learning is not sufficient to explain all forms of animal learning. Studies have shown that rats and birds are capable of learning sequences of events and they can use this knowledge to predict future rewarding events that have yet to be experienced (Clayton et al., 2003; Jones et al., 2012). Furthermore, in social learning experiments, animals can learn from others by observing their decisions and the resulting outcomes, and adjust their own actions without having directly experienced the outcomes themselves (Subiaul et al., 2004; Monfardini et al., 2012). Principles analogous to those driving reinforcement learning may be involved in these cases, including the updating of expectations based on sensory inputs, but these types of learning require additional computational components besides feedback from outcome (Camerer, 2003; Montague, 2007; Seo and Lee, 2008). Computationally, this may include a module for observing what happens to others and for adjusting one's own preferences based on these observations. The brain areas involved in these processes are under active investigation (Behrens et al., 2008; Suzuki et al., 2012).

These findings indicate that animals, including humans, can learn without direct experience. The mechanisms by which this type of learning occurs are very diverse, and may include both simple enhancement of attention to others, in the case of socially facilitated food preferences, and the recognition of emotional facial cues in others as they experience outcomes, to more complex mechanisms including mentalizing and theory of mind.

## Overview of neural circuits implicated in social learning in humans

A number of studies have implicated specific brain areas in human social behavior. These areas include the temporoparietal junction (TPJ), the anterior cingulate gyrus (ACCg), the dorsomedial prefrontal cortex (DMPFC), and the dorsolateral prefrontal cortex (DLPFC). All of these regions may contribute to the interpretation of others' intentions and social learning (Behrens et al., 2009). The TPJ integrates systems for memory, language, attention, and social processing and its activation is correlated with the degree to which an opponent is perceived as intelligent (Carter and Huettel, 2013). Moreover, gray matter volume in the TPJ predicts altruistic tendencies (Morishima et al., 2012). TPJ has been implicated in mentalizing and understanding intentions, suggesting involvement in empathy, altruism, and learning or strategizing in a competitive context (Samson et al., 2004; Carter et al., 2012). By contrast, the dorsolateral prefrontal cortex (dlPFC) may contribute to executive control, planning, and goal-directed behavior in social contexts, particularly deception (Miller and Cohen, 2001; Knoch et al., 2006). The dorsomedial prefrontal cortex underlies processes including cognitive control and social interaction (Venkatraman et al., 2009). Studies of the anterior cingulate gyrus (ACCg) have revealed involvement in error correction and reinforcement learning from social outcomes as well as emotional and facial expression recognition (Behrens et al., 2008; Venkatraman et al., 2009; van den Stock et al., 2013).

**Figure 1:**
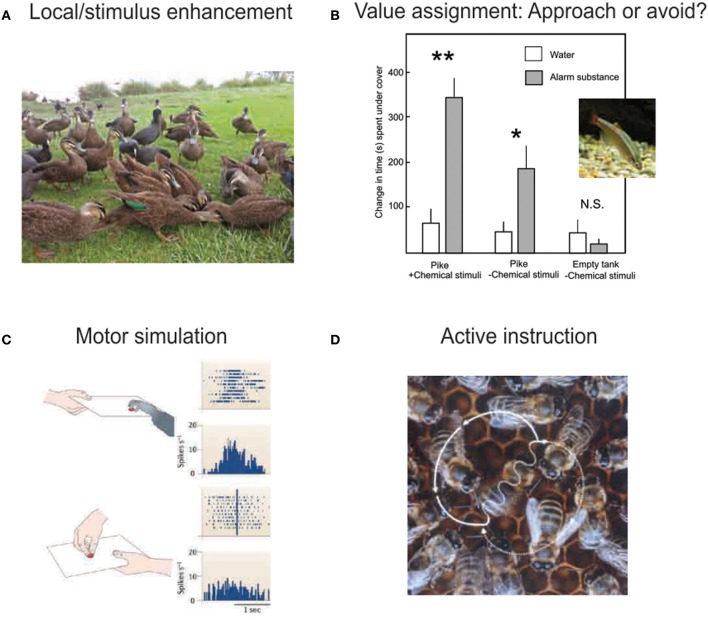
**Socially facilitated learning occurs through a variety of mechanisms. (A)** By drawing attention to a particular location or object, social cues make foraging-relevant features more salient. Such cues may or may not be intentionally delivered by the signaler. Birds commonly use flocking information to identify the location of a food patch. Image by Dan Knudson. **(B)** Signals released or displayed by other individuals, including approach or avoidance behaviors, facial expressions, and chemical deposits, signal the valence of the enhanced stimulus or location. Here, minnows spend more time undercover in response to a predator the initial exposure to the predator is paired with alarm substance. Bars indicate increase in time spent hiding after a training exposure to a pike with (open bars) or without (gray bars) alarm substance. Measurements are taken during exposure to pike and alarm substance, pike without alarm substance (water only), or empty tank without alarm substance, 1, 3, and 5 days after initial exposure, respectively. ^*^P < 0.05; ^**^P < 0.01. Figure modified with permission from (Chivers and Smith, 1994). Minnow image by Sanse, via Wikimedia Commons. **(C)** Although few non-human species have been found to imitate other individuals in the strict sense, the observation and performance of motor behaviors are known to activate overlapping neural circuitry. “Mirror neurons” in the frontal cortex of macaque monkeys fire both when performing a motor act and when watching another individual perform the act. This could provide a mechanism by which appropriate behavior is “primed” in a naive individual that observes a knowledgeable conspecific. Figure reproduced with permission from (Iacoboni and Dapretto, 2006). **(D)** In the process of active instruction, specific information is intentionally communicated to other individuals. This is known to occur in the context of the bee waggle dance, in which the travel path to a remote nectar site is signaled to other foragers in the hive. Image by J. Tautz and M. Kleinhenz, Beegroup Würzburg, via Wikimedia Commons.

In this review, we will explore current knowledge on the contexts in which social learning occurs in non-human animals and the brain mechanisms underlying such forms of learning. Social learning can happen through a variety of mechanisms that may include effects of others on attention (Figure 1A), learning stimulus or action value through observation (Figure 1B), motor simulation and imitation (Figure 1C) and active instruction using movements or sounds (Figure 1D). The brain substrates that mediate these skills often subserve non-social cognitive and motivational processes as well. Based on these observations, we hypothesize that many cognitive and motivational systems that originally evolved to solve non-social problems have been co-opted by evolution to contend with social challenges (Gould and Lewontin, 1979). Complementing these general-purpose mechanisms are a small set of brain areas for which there is tantalizing evidence of uniquely specialized social functions, which may have evolved in only a limited number of species that have confronted the most complex social environments. These potentially uniquely social mechanisms remain to be fully described, in part due to the difficulty of studying them in standard model animal species that often lack the extreme social complexity found in humans, some great apes, and highly social birds like corvids.

## Transmission of reward information during group foraging

Many animal species forage in groups. Individuals in those groups may obtain information on food location from the behavior of their fellow group members. Foraging in groups has been proposed to increase the probability of finding food through an effect referred to as local enhancement. Local enhancement is the benefit that an animal obtains from being in a flock by having multiple members scanning the environment, thus increasing the likelihood of finding food (Krebs et al., 1972; Beauchamp, 1998). The discovery of a food patch in a location in space (local enhancement) or associated with a particular cue (stimulus enhancement) attracts the attention of the other group members, a phenomenon well documented in birds (Spence, 1937; Krebs et al., 1972; Brown, 1986; Krebs and Inman, 1992; Avery, 1994) (Figure 1A). Roosts and colonies of birds may also fill the role of information centers, in which individuals identify the most successful foragers and follow them to food sources (Brown, 1986; Rabenold, 1987; Bugnyar and Heinrich, 2005). Bats, which rely on echolocation to hunt, are attracted to playbacks of echolocation calls produced during prey capture, suggesting that social information can guide individuals to successful hunting sites (Dechmann et al., 2009). It has also been shown in three species of titmice that social network size influences the likelihood of discovering novel food patches, suggesting that there is an evolutionary benefit to developing a larger network of social connections (Aplin et al., 2012). Rats leave scents at sites where novel, attractive food has been found, which subsequently serves as a guide for other rats to locate the sites. This phenomenon suggests that olfactory cues can transmit information about food sources as well (Galef and Beck, 1985). In addition, worker honeybees receiving sugar in hives from incoming foragers learn to associate floral odors with behavioral responses as the foragers transfer the sugar (Farina et al., 2007). Finally in some species, including ravens and chimpanzees, the individuals finding a food patch can emit vocal signals that attract other members of their group (Heinrich, 1988; Slocombe and Zuberbühler, 2006).

### Attention to others

Although there is strong evidence that animals are influenced by others' foraging activities, the neural mechanisms by which individuals gather information from others remain unknown in the majority of cases, due to the technical difficulties inherent in applying neurophysiological techniques in the wild. Some studies have succeeded at creating laboratory experiments that recapitulate specific aspects of interactions that may happen during group foraging. In the laboratory, monkeys are known to be powerfully attracted to photos of other individuals, and this may reflect an important building block of social attention that makes other individuals interesting stimuli for animals (Deaner et al., 2005). The orbitofrontal cortex might be an important piece of the network allocating such social attention as it carries signals related to the value of gustatory rewards as well as signals related to the social influence and attentional priority of other individuals (Watson and Platt, 2012). Likewise the lateral intraparietal area signals the value of social information for choosing where to look (Klein et al., 2008, 2009; Klein and Platt, 2013). The TPJ has also been shown to be involved both in attentional processes (Corbetta and Shulman, 2002) and social cognition (Saxe and Kanwisher, 2003); thus it could constitute an important node for orienting attention to others during foraging. Evidence from connectivity analyses suggest that the TPJ is composed of subregions with distinct connectivity profiles, some regions showing activities correlated with other parts of the brain involved in social cognition and/or attention (Mars et al., 2012; Bzdok et al., 2013). The specific role of these subregions in attention and social cognition remains to be explored. Vocalizations related to food and social relationships have been shown to activate regions of the temporal lobe in macaques, which may play a role in identifying the meaning of the calls and drawing attention to others in critical situations (Gil-da-Costa et al., 2004). Although their involvement in natural group foraging contexts is only speculative at the moment, these areas may contribute to orienting gaze toward other individuals, and may constitute the building blocks of the neural systems that direct attention to others and potentially carry out neural computations that contribute to social influences on foraging.

### Gaze-following

Group foraging may also rely on extracting finer information from others, such as where they are looking, a phenomenon known as gaze-following or joint attention. The superior temporal sulcus (STS) (Kamphuis et al., 2009; Laube et al., 2011) and amygdala (Emery, 2000; Tazumi et al., 2010; Gordon et al., 2013), in monkeys and humans, respond to the sight of other individuals orienting in a particular direction. Further, impaired amygdala function in monkeys and humans disrupts gaze-following behavior (Kennedy and Adolphs, 2010; Roy et al., 2012). In macaques, the activity of neurons in the lateral intraparietal area—a brain region implicated in attention and orienting—is modulated by the gaze of others, a potential mechanism for directing attention to objects and locations attended by them (Shepherd et al., 2009). In humans, the gaze of others influences where people look and may even change their perception of objects (Ricciardelli et al., 2002; Frischen, 2007). Much remains to be discovered to understand these effects, but brain imaging studies demonstrate that some areas, including the dorsal striatum, anterior cingulate and inferior frontal cortex, show differential activation when individuals track the gaze of others (Schilbach et al., 2011). Thus, there are mechanisms in the brain that track the actions of others and the objects of their attention, but how these mechanisms are integrated to guide foraging decisions remains almost completely unknown.

### Outcome monitoring

Learning from the foraging choices of others also requires neural processes that encode information relating to rewards and which individuals have obtained them. For example, neurons in the dorsal anterior cingulate cortex (ACCs) respond to missed opportunities, including rewards received by others (Hayden et al., 2009; Chang et al., 2011), whereas neurons in the anterior cingulate gyrus selectively signal the rewards received by others (Chang et al., 2013). Other areas of the brain are known to play roles in learning and reward-guided decision-making. In particular, the ventromedial prefrontal cortex (Kolling et al., 2012), ventral striatum (Klimecki et al., 2013) and dopaminergic midbrain (Schultz et al., 1997) all play important roles in reinforcement learning and motivation in non-social contexts. The ventral striatum has been shown to be modulated by expectations developed when learning in a social context, suggesting that part of the brain networks involved in social learning may overlap with the networks responsible for learning from direct experience (Jones et al., 2011). These data suggest that the brain areas involved in social influences on attention and food consumption by others overlap with areas involved in cognition and motivation in non-social context.

## Transmission of preferences

**Figure 2:**
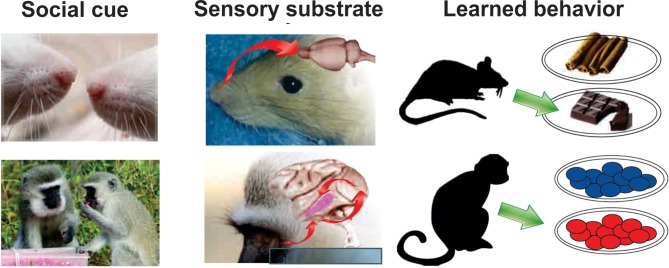
**Sensory modalities underlying social learning differ across species.** In socially-facilitated food preference, rats (top) rely heavily on olfactory signals. Olfactory trails laid by conspecifics can signal what to eat and where to find it. Moreover, olfactory components from the food detected on the breath of a conspecific, causes rats to prefer the associated food, even when tested weeks later. This preference can also be elicited by pairing the food odorant with carbon disulfide, a volatile chemical found in the breath (reviewed in Galef, 2012). In contrast, primates (bottom) are heavily visual. Visual cues convey information about the food as well as about the social agent associated with the food. Social information such as kin relationship, rank, and group membership modulates the effect of social cues on food-related learning (van de Waal et al., 2013). Rat noses photo by Alexey Krasavin; rat nose photo by Robin Stjerndorff; Chocolate photo by Simon A. Eugster; Cinnamon photo by trophygeek; Vervet head photo by Wegmann, all from Wikimedia Commons. Brain photos courtesy of University of Wisconsin and Michigan State Comparative Mammalian Brain Collections. Vervet food sharing photo modified from (van de Waal et al., 2013), with permission.

Beyond sharing information about the location of resources, animals may also learn about the quality of specific foods from others. In humans, eating habits in children are strongly influenced by familial and social factors (Patrick and Nicklas, 2005), and adults' food preferences are modulated by those of their dining companions (Young et al., 2009). In macaques, when mothers develop an aversion to specific foods, this results in reduced consumption of those foods by infants (Hikami et al., 1990). Infant vervet monkeys and males that immigrate to new social groups conform to local food preferences (Figure 2) (van de Waal et al., 2013). In rats, social transmission of food avoidance behavior is present and depends on the learner's previous exposure to food to be avoided (Masuda and Aou, 2009).

### Fear responses

One of the most studied types of preference transmission is learning what to fear by observing others (Olsson and Phelps, 2007). Many animal species are capable of learning to fear a stimulus by observing the behavior of another animal toward it, including sheep (Keller et al., 2004), rats (Kavaliers et al., 2001), cats (John et al., 1968), monkeys (Cook and Mineka, 1989), mice (Jeon et al., 2010), and humans (Gerull and Rapee, 2002). The amygdala is a candidate site for this type of learning due to its known role in fear responses learned from direct experience (Olsson and Phelps, 2007). Functional magnetic resonance imaging studies have shown that the amygdala is activated during observational fear learning in humans (Hooker et al., 2006; Olsson et al., 2007). Furthermore, amygdala damage impairs fear recognition by disrupting the ability to use information from the eye region of faces (Adolphs et al., 2005). In addition, recent evidence indicates that disruption of activity in the anterior cingulate cortex of mice impairs observational fear learning (Jeon et al., 2010).

### Quality of food

Theoretically, one can learn the preferences of others by observing their attraction to good outcomes or by avoidance of bad outcomes (Figure 1B). Different mechanisms can be at play in any animal species and specific experimental context. The studies by Hikami et al. (1990) and Masuda and Aou (2009) used avoidance and disgust reactions to transmit food preferences. In domestic hens, learning to avoid foods was not observed in experimental conditions, but the frequency of pecking of good food did increase the proportion of food eaten by observers (Sherwin et al., 2002). This suggests that the transmission of preferences may rely on good or bad experiences depending on learning context (Sherwin et al., 2002).

The brain systems that permit animals to observe outcomes that occur to others and transform these observations into appropriate decisions are still under investigation. Chang and colleagues showed that deciding to give rewards and viewing another monkey receive a reward activate the same subset of neurons in the anterior cingulate gyrus. In comparison, activity in the orbitofrontal cortex is selective for rewards delivered to self and activity in the anterior cingulate sulcus is selective for foregone rewards (Chang et al., 2013). In rats, it has been shown that cholinergic neurotransmission in the orbitofrontal cortex is necessary for social learning of food preferences (Ross et al., 2005). These findings suggest that the anterior cingulate gyrus and orbitofrontal cortex may be specialized for processing information about the experiences of others, but how this information is translated into modifications of behavior during social learning is poorly understood.

### Identity and tutoring

Individuals vary in whom they trust for information to guide learning (Coussi-Korbel and Fragaszy, 1995). Important social factors include identity and characteristics of the demonstrator. There is a strong correlation between the number of other individuals engaging in a behavior and an individual's likelihood of replicating the behavior or otherwise conforming (Galef and Laland, 2005). In addition, familiarity is an important modulator of social learning, as humans and other animals are more likely to learn from familiar individuals than from strangers. This phenomenon can be observed across species. For example, guppies learned a swimming route to food significantly faster when the demonstrator was familiar to them (Swaney et al., 2001). Expertise also modulates learning, with naïve chimpanzees spending more time following successful or informed conspecifics than other naïve chimps (Menzel, 1974; Galef and Laland, 2005). Age can also affect learning; in particular juveniles can learn from adults (Galef and Laland, 2005; van de Waal et al., 2013). In one study, juvenile rats only ate foods they had observed elders eating previously and sampled food from the mouths of elders to acquire food preferences whereas elders sampled food from juveniles significantly less frequently (Galef and Giraldeau, 2001). It has been shown that in small-scale human societies, children ages 10 and up prefer to learn from others perceived as more successful/knowledgeable and that age and sex also influence who is picked as tutors (Henrich and Broesch, 2011). Finally, dominance ranking modulates social learning. For example, hens learn more effectively from dominant hens than from unfamiliar or subordinate ones (Nicole and Pope, 1999). How identity modulates social learning varies across species. For instance, it has been reported that chimpanzees use information from older adults to learn unusual feeding behaviors, whereas gorillas learn preferentially from younger individuals (Masi et al., 2012). Therefore, the influence of identity and expertise on social learning is a widespread phenomenon in animals although the specific characteristics of the individuals likely to improve social learning varies across species.

Given the influence of identity on social learning, it is interesting to examine the brain areas that may process such information. The effects of familiarity on social learning may be mediated by brain regions that process identity information encoded in faces, including the fusiform face area (Haxby et al., 2002) and along the gyral surface of the temporal lobe (Tsao et al., 2008; Freiwald and Tsao, 2010). Increases in social network size in macaques are associated with increases in gray matter in mid-superior temporal sulcus and rostral prefrontal cortex (Sallet et al., 2011). Cells in the prefrontal cortex have been shown to be modulated differently according to dominance and social context (Fujii et al., 2009). Using functional magnetic resonance imaging, two neighboring divisions of the anterior cingulate cortex were found to encode variables related to direct experience learning and learning from social information separately (Behrens et al., 2008). This study employed a simple decision task in which participants could base their decisions on their own experience or on the suggestions of a confederate, each of which could be modeled orthogonally. Behrens et al. (2008) proposed that social value could be subject to an associative learning process similar to that applied to other non-social stimuli. For instance, by registering the advice of the confederate and computing a prediction error with respect to current knowledge, one could determine the trustworthiness of the confederate. The activity of three regions of the brain was shown to correlate with this computation: the anterior cingulate cortex gyrus, the temporoparietal junction, and the dorsomedial prefrontal cortex (Behrens et al., 2008). These findings suggest that these areas might be involved in the processes by which an individual learns about the reliability of others' advice. This possibility relates to the ability of humans and other animals to focus on learning from an informed expert over a naïve conspecific. It has been shown that macaques prefer viewing dominant individuals (Deaner et al., 2005). Social hierarchy is associated with modulations of the ventral striatum and amygdala in humans (Zink et al., 2008; Ly et al., 2011; Kumaran et al., 2012) and the medial prefrontal cortex plays a causal role in dominance-related behaviors in mice (Wang et al., 2011). These networks seem to encode information about the identity of those with whom a given individual interacts and therefore could constitute the neural basis for the influence of identity on social learning.

### Emotion recognition and empathy

The recognition of facial and behavioral expressions of fear and disgust is another mechanism by which individuals may learn from the experiences of others. It has been shown that the anterior cingulate cortex and frontoinsular cortices are activated by fearful facial expressions, suggesting that these regions might process social information associated with negative outcomes (Fan et al., 2011). The ventromedial, dorsomedial, and dorsolateral prefrontal cortex may also be involved in tracking the decisions of others since these regions encode the reward and action prediction errors obtained from observing others' decisions (Behrens et al., 2008; Suzuki et al., 2012). In macaques, dynamic facial expressions increase BOLD signal in the anterior superior temporal sulcus (Furl et al., 2012). The amygdala and dorsal anterior cingulate cortex also appear to be involved in self-monitoring of social facial expressions (Livneh et al., 2012). Amygdala lesions also change the activation patterns of the inferior temporal cortex in response to facial expressions (Hadj-Bouziane et al., 2012). These findings suggest that an extended brain system processing facial expressions is present in macaques (Tsao et al., 2008; Freiwald and Tsao, 2010). It remains to be determined if the facial recognition skills of primates are necessary for social learning of food preference and fear association or whether other behavioral signs are used to recognize positive and negative emotions in others.

A role for the ACC in empathy is supported by imaging studies in humans showing that this area responds to pain felt by others (Singer et al., 2004; Bernhardt and Singer, 2012). The anterior insula also seems to respond strongly to viewing others in pain (Singer et al., 2004; Gu et al., 2010). Furthermore, lesion studies indicate that both ACC and insula lesions can contribute to reductions in affective empathy (Leigh et al., 2013). Theory of mind, the cognitive processes by which people model the goals, intentions and emotions of others, is thought to rely on a wide network of brain regions including the superior temporal sulcus, temporo-parietal junction, precuneus, and the medial prefrontal cortex (Koster-Hale and Saxe, 2013). Therefore, understanding others and sharing their emotions relies on an extended brain network with components in the prefrontal, parietal, and temporal cortices.

### Olfactory cues

A body of work initiated by Bennett Galef over 40 years ago demonstrates that, even within a single species, food choices are biased by many distinct social mechanisms that operate via different modalities. For example, lactating mother rats, like humans (Mennella, 1995), transmit taste preferences to their offspring via milk flavor (Galef and Clark, 1972; Galef and Henderson, 1972). In the olfactory domain, rats follow scent trails of other rats to food sites (Galef, 1996), and to prefer food deposits scent-marked by other rats (Galef and Heiber, 1976). In the visual domain, young rats leaving the nest learn to locate food sites by visually identifying the location of adult rats (Galef and Clark, 1971). In this last example, the visual cue is sufficient for learning, and the presence of an anesthetized or dead adult rat elicits similar spatial orientating behavior.

In a particularly striking example of social learning, Galef also discovered that food preferences are socially transmitted between rats at points that are temporally and spatially distant from the food source, in a manner somewhat analogous to humans seeking restaurant recommendations from friends (Figure 2). Galef found that, after “demonstrator” rats ate cocoa-flavored rat chow, young “observer” rats preferred cocoa-laced rat chow over cinnamon-laced rat chow after interacting with the demonstrator (Galef, 2003; Galef and Whiskin, 2003). The cue responsible for this preference was subsequently found to be olfactory, as exposure to rat breath laced with cocoa, or even human breath laced with cocoa, could induce this preference in observer rats (Galef, 2009). Even more specifically, the presence of carbon disulfide, a gas present in rat breath, when paired with cocoa, was found to be sufficient to induce food preference, as a stuffed dummy rat laced with cocoa, while insufficient on its own to induce preference, would induce preference when laced with cocoa paired with a few drops of carbon disulfide. The ability to detect flavors depends on a signaling cascade initiated by guanylyl cyclase-expressing olfactory receptors in the nasal epithelium, and mouse knock-outs of the genes encoding these receptors show no preference for the flavor consumed (Munger et al., 2010).

Social learning of food preferences is not limited to mammals and birds. Some species of fish, including fathead minnows, have specialized epidermal cells that release “alarm substance” when mechanically damaged. This chemical alarm substance diffuses through the water to enhance predator escape responses amongst the surrounding individuals (Göz, 1942; Chivers and Smith, 1994; Griffin, 2004). Alarm substance can be viewed as analogous to carbon disulfide in the breath of conspecifics in the case of rats, though in rats the chemical induces approach behavior and in fish the chemical induces avoidance (Figure 2).

Socially-induced food preferences are long-lasting, known to last for weeks after exposure to the demonstrator. Lesburguères found that long-term memory of a socially induced food preference is mediated by connections relying on NMDA/AMPA receptors between the hippocampus and orbitofrontal cortex (OFC) (Lesburguères et al., 2011). They posit that such memories retain their specificity for the preferred food using an epigenetic tagging mechanism, in which specific neurons in the OFC are designated at the time of exposure as the ultimate carriers of this memory, even though it will be days before the memory gets consolidated. Ross and Eichenbaum (2006) have shown that damage to the hippocampus in rats impairs social transmission of food preferences. How the brain integrates social cues to shape future choices remains to be investigated but the mechanisms may include computations of the difference between one's own preferences and the preferences of others, and integration of the identity of others, a variable that correlates with activity in the dorsomedial prefrontal cortex (Izuma and Adolphs, 2013).

Current studies thus provide a rough picture of the brain areas that may be involved in tracking the valence of outcomes occurring to others. As shown in the previous section, social learning of preferences may rely on simple mechanisms such as favoring attention to where others are looking. In addition, social learning may rely on recognizing whether an outcome is good or bad. One important challenge for future research will be to identify the neural mechanisms by which these processing streams influence decision-making. Given the fact that social learning can rely on various sensory inputs including vision, audition and olfaction, the brain mechanisms underlying social learning in the wide array of species that show this ability may be very different. Among the most interesting questions to explore is whether or not the brain systems mediating socially-learned preferences overlap with the brain systems mediating non-socially learned preferences.

## Transmission of skills, actions, and goals

Animals are also capable of learning new skills, foraging methods, and social conventions by observing conspecifics (Figures 1C,D). The potato-washing and wheat-winnowing behaviors of Japanese monkeys are among the most well-known examples. Kawamura (1959) observed the propagation of these behaviors from individuals to their relatives and friends, and then to the extended group. In wild meerkats, naïve pups are more likely to consume food that requires handling skills, such as hardboiled eggs and scorpions, if they are given the opportunity to observe an adult eating those foods (Thornton, 2008). A long-term study looked at traditions or social conventions in white-faced capuchin monkeys, defining those as behaviors that are common in subpopulations of capuchin monkeys while absent among other populations, implicating social influences on learning (Perry et al., 2003). Several behaviors were found to qualify as traditions or social conventions, including hand-sniffing, sucking of body parts, and playful gestures displayed with another individual (Perry et al., 2003). In populations of white-faced capuchin monkeys, young foragers can observe and learn from mature foragers who consume food requiring multi-step processing (Perry, 2011). Learning skills from others occurs in a wide range of other animals as well, including octopuses, birds, and mammals (Sherry and Galef, 1984; Fiorito and Scotto, 1992; Thornton, 2008). Chimpanzees and humans also demonstrate impressive abilities to learn complex sequences of actions through observation (Whiten et al., 1996; Whiten, 1998). Chimpanzees have been shown to transmit to others nut-cracking techniques involving stones or tree roots and ant-dipping through direct mouthing and pull-through (Humle and Matsuzawa, 2002; Humle et al., 2009; Luncz et al., 2012). Much remains to be discovered concerning the neural mechanisms underlying such cultural transmission of behavior, but a study on communicative innovation has identified activation in the ventromedial prefrontal cortex and the temporal lobe when pairs of human subjects generate and subsequently understand novel communicative symbols (Stolk et al., 2013).

### Imitation and emulation

Emulation and imitation are forms of social learning in which individuals actively model the goal of another individual's actions (Wood, 1989; Tomasello et al., 1993; Horner and Whiten, 2005). In emulation, the observer only gathers information about the goal that is attained by the observed individual but independently learns the appropriate actions to reach the identified goal, typically by trial and error. In imitation, the observer not only emulates the goal, but also the sequence of actions to reach that goal.

Cognitive imitation is a subset of imitative behaviors. Subiaul et al. (2004) showed that macaques are capable of learning to touch sequences of images in order to reach a reward, independent of the precise sequence of actions needed. In this case, learning is abstract (image sequence) rather than physical (actions performed), hence the term “cognitive imitation.” There remains active research on the specific learning contexts that involve either emulation or imitation in humans and chimpanzees. There is strong evidence that chimpanzees can successfully observe actions and reproduce certain aspects of the performed actions, and the phenomenon has been referred to as imitation by some authors (Whiten et al., 1996; Bjorklund et al., 2000; Myowa-Yamakoshi et al., 2004; Bard, 2007; Carrasco et al., 2009). However, other authors have shown that chimpanzees fail to imitate novel actions. They argue that the majority of devices utilized in social learning experiments can lead the subject to copy by process of emulation, and therefore chimpanzees may in fact learn the physical movements of these devices, rather than the actions of another individual (Call et al., 2005; Tennie et al., 2012).

**Figure 3:**
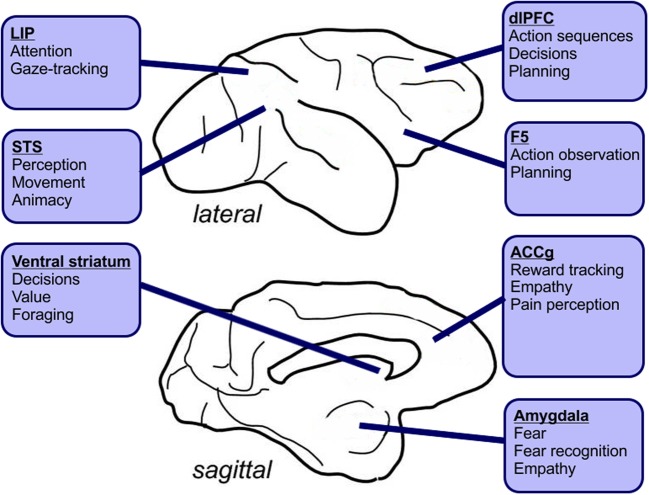
**Hypothetical roles for macaque brain areas known to be involved in social interactions, planning and perception.** Social learning may involve directing attention at others or tracking their gaze. It may also involve observing their behaviors and emulating or imitating sequences of actions. Finally, some forms of social learning might rely on observing outcomes, preferences and aversion or fear. LIP, Lateral intraparietal area; STS, Superior temporal sulcus; dlPFC, Dorsolateral prefrontal cortex; ACCg, Anterior cingulate cortex gyrus.

Despite the “emulation vs. imitation” debate, it remains necessary to outline possible neural circuits that may be involved in learning skills through observation. For emulation, an act as simple as diverting the learner's attention to the goal of others may be sufficient to favor learning. Additionally, for both emulation and imitation, skill learning often involves sequential behaviors; do A, then B, followed by C. Research in the past few decades has revealed brain areas that may be involved in processing such action sequences. Decision-making and the performance of sequences of behaviors are likely complex processes involving continuous adjustments of attention, goals, and motor plans (Figure 3) (Resulaj et al., 2009). Using fMRI, it has been shown that the brain areas active during the inhibition of imitative responses in humans overlap with those involved in mental state attribution, specifically the TPJ and anterior fronto-median cortex, frontal gyrus and superior parietal lobule (Buccino et al., 2004; Brass et al., 2009; Caspers et al., 2010). It has also been shown using trans-cranial magnetic stimulation to disrupt the right TPJ that this area plays a causal role in imitation (Sowden and Catmur, 2013).

The contributions of other areas remains speculative for the moment because it is hard to create laboratory contexts in which animals repeatedly learn socially, but many experiments in which animals learn sequences of actions non-socially permit us to sketch the potential role of prefrontal areas in learning sequences of movements. For instance, neurons in the anterior cingulate cortex are activated differentially based on the number of instances in which an action was repeated in a sequence (Iwata et al., 2013). Neurons in the lateral prefrontal cortex are modulated by action sequences and fire spikes for specific sequences of actions, rather than individual actions (Shima et al., 2007; Tanji and Hoshi, 2008). Neurons in the pre-supplementary motor area also encode temporal aspects of behavioral sequences (Shima and Tanji, 2006; Lucchetti et al., 2012), and fMRI signals from this region in humans also respond to ordering tasks (Acuna et al., 2002). By activating GABA receptors with muscimol injections, a procedure that inhibits the activity of neurons of a specific brain area, it has been found that both the supplementary and pre-supplementary motor areas were necessary to perform normally on memory-based sequences of movements (Shima and Tanji, 1998). The anterior cingulate cortex, supplementary and pre-supplementary motor areas, and lateral prefrontal cortex thus appear to be potential candidates for components of the network required to learn skills from others given their role in encoding and processing sequences of actions. However, the direct involvement of these areas in the social learning of skills has yet to be tested.

### Action observation and mirror neurons

Observing sequences of actions is a necessary initial step to extracting information from others and learning from them (Bonini et al., 2013). One proposed mechanism through which this may occur is the mirror neuron system, although this proposition is highly debated (Newman-Norlund et al., 2007; Hickok, 2009). Mirror neurons were first described in monkeys as cells that fire both when an animal performs an action and observes another animal performing the same action (di Pellegrino et al., 1992). In monkeys, these cells are found in the prefrontal cortex area F5 (di Pellegrino et al., 1992) and in the parietal cortex (Fogassi et al., 2005; Rozzi et al., 2008). In humans, functional magnetic resonance imaging has revealed a set of areas that are activated when subjects view grasping actions of others, including the ventral premotor cortex, posterior frontal gyrus, and inferior frontal gyrus (Iacoboni et al., 2005). Differences arise between activation of these regions of the brain when monkeys and humans view an identical action in different contexts, which suggests that neurons in these areas encode aspects of the action's goal and context, which could indicate a role in intention understanding (Fogassi et al., 2005; Iacoboni et al., 2005).

Other studies have identified cells in the medial frontal cortex that respond to other's actions separately from self-actions (Yoshida et al., 2011). Furthermore, neurons in this area respond to observing errors made by others (Yoshida et al., 2012). These findings suggest a potential role for the medial frontal cortex in monitoring social outcomes. Both the ventral premotor cortex and the parietal cortex contain neurons that respond both to the actions of others and to one's own actions (Fujii et al., 2008), and these responses are modulated by the presence of food that both monkeys can grab (Fujii et al., 2007). The frontal and parietal networks that contain mirror neurons are linked to each other by numerous connections in macaques, chimpanzees and humans (Hecht et al., 2013). Independent subdivisions of the medial prefrontal cortex are active when one makes choices for oneself or for a partner, suggesting that actions made by oneself and others are represented separately in the media prefrontal cortex (Nicolle et al., 2012). It remains unknown whether or not mirror neurons and the brain areas showing mirror-like hemodynamic responses in fMRI studies causally contribute to social learning. Thus, one of the challenges for future research will be to identify learning contexts in which these areas are necessary for social learning to occur. To accomplish this goal, setups will be required in which social learning can occur consistently in a laboratory setting, in conjunction with local manipulation of groups of neurons in the prefrontal and parietal cortex. The currently available data indicates that the actions of self and others can be represented jointly in some brain areas while separately in others, and that many of the areas involved in social learning also have roles in non-social learning.

## Conclusion

The contexts in which social learning and social influences on learning occur are numerous, and these skills are found in a broad range of species. However, the neural mechanisms underlying these skills remain poorly understood. In some cases, even the precise cues used by individuals to extract social information remain unknown. Social learning occurs when sensory inputs generated by others are used as sources of information by decision-makers. Most of the cases reviewed here involve learning from conspecifics, but there are known cases of interspecies social learning, including in elephants and parrots (Balsby et al., 2012; Stoeger et al., 2012). To investigate social learning, it will be necessary to identify the sensory cues that allow individuals to learn socially in a broad range of species. Visual (facial expression recognition, behavioral recognition), auditory (screams, food consumption sounds), and olfactory (smell of another's animal breath) cues are all distinct possibilities. The second challenge will be to develop a variety of animal models that allow for experimental manipulations of these cues in order to characterize the role of different brain processes in social learning. Recording neurons and manipulating the activity of specific brain areas while social learning occurs will be necessary to reveal the processes that mediate social learning. Ultimately, how the brain processes social information will be crucial in our understanding of human social interactions and culture, and may suggest new ways to treat neuropsychiatric disorders attended by impaired social interactions, as well as the development of enhanced educational methods.

### Conflict of interest statement

The authors declare that the research was conducted in the absence of any commercial or financial relationships that could be construed as a potential conflict of interest.

## References

[B1] Acuna B. D. Eliassen J. C. Donoghue J. P. Sanes J. N. (2002). Frontal and parietal lobe activation during transitive inference in humans. Cereb. Cortex 12, 1312–1321 10.1093/cercor/12.12.1312 12427681

[B2] Adolphs R. Gosselin F. Buchanan T. W. Tranel D. Schyns P. Damasio A. R. (2005). A mechanism for impaired fear recognition after amygdala damage. Nature 433, 68–72 10.1038/nature03086 15635411

[B3] Aosaki T. Tsubokawa H. Ishida A. Watanabe K. Graybiel A. M. Kimura M. (1994). Responses of tonically active neurons in the primate's striatum undergo systematic changes during behavioral sensorimotor conditioning. J. Neurosci. 14, 3969–3984 8207500 10.1523/JNEUROSCI.14-06-03969.1994 PMC6576948

[B4] Aplin L. M. Farine D. R. Moran-Ferron J. Sheldon B. C. (2012). Social networks predict patch discovery in a wild population of songbirds. Proc. R. Soc. B 279, 4199–4205 10.1098/rspb.2012.1591 22915668 PMC3441092

[B5] Avery M. L. (1994). Finding good food and avoiding bad food – does it help to associated with experienced flockmates? Anim. Behav. 48, 1371–1378 10.1006/anbe.1994.1373

[B6] Balsby T. J. Momberg J. V. Dabelsteen T. (2012). Vocal imitation in parrots allows addressing of specific individuals in a dynamic communication network. PLoS ONE 7:e49747 10.1371/journal.pone.0049747 23185424 PMC3504101

[B7] Bard K. A. (2007). Neonatal imitation in chimpanzees (Pan troglodytes) tested with two paradigms. Anim. Cogn. 10, 233–242 10.1007/s10071-006-0062-3 17180698

[B8] Beauchamp G. (1998). The effect of group size on mean food intake rate in birds. Biol. Rev. 73, 449–472 10.1017/S000632319800524619070065

[B182] Behrens T. E. Hunt L. T. Rushworth M. F. (2009). The computation of social behavior. Science 324, 1160–1164 10.1126/science.1169694 19478175

[B9] Behrens T. E. Hunt L. T. Woolrich M. W. Rushworth M. F. (2008). Associative learning of social value. Nature 456, 245–249 10.1038/nature07538 19005555 PMC2605577

[B10] Bernhardt B. C. Singer T. (2012). The neural basis of empathy. Annu. Rev. Neurosci. 35, 1–23 10.1146/annurev-neuro-062111-150536 22715878

[B11] Bjorklund D. F. Bering J. M. Ragan P. (2000). A two-year longitudinal study of deferred imitation of object manipulation in a juvenile chimpanzee (Pan troglodytes) and orangutan (Pongo pygmaeus). Dev. Psychobiol. 37, 229–237 10.1002/1098-2302(2000)37:4%3C229::AID-DEV3%3E3.0.CO;2-K 11084604

[B12] Bonini L. Ferrari P. F. Fogassi L. (2013). Neurophysiological bases underlying the organization of intentional actions and the understanding of others' intention. Conscious. Cogn. 22, 1095–1104 10.1016/j.concog.2013.03.001 23545395

[B191] Brass M. Ruby P. Spengler S. (2009). Inhibition of imitative behaviour and social cognition. Philos. Trans. R. Soc. Lond. B Biol. Sci. 364, 2359–2367 10.1098/rstb.2009.0066 19620107 PMC2865080

[B13] Brown C. R. (1986). Cliff swallow colonies as information centers. Science 234, 83–85 10.1126/science.234.4772.83 17742636

[B190] Buccino G. Vogt S. Ritzl A. Fink G. R. Zilles K. Freund H. J. (2004). Neural circuits underlying imitation learning of hand actions: an event-related fMRI study. Neuron 42, 323–334 15091346 10.1016/s0896-6273(04)00181-3

[B14] Bugnyar T. Heinrich B. (2005). Ravens, Corvus corax, differentiate between knowledgeable and ignorant competitors. Proc. Biol. Sci. R. Soc. 272, 1641–1646 10.1098/rspb.2005.3144 16087417 PMC1559847

[B15] Bzdok D. Langner R. Schilbach L. Jakobs O. Roski C. Caspers S. (2013). Characterization of the temporo-parietal junction by combining data-driven parcellation, complementary connectivity analyses, and functional decoding. Neuroimage 81, 381–392 10.1016/j.neuroimage.2013.05.046 23689016 PMC4791053

[B16] Call J. Carpenter M. Tomasello M. (2005). Copying results and copying actions in the process of social learning: chimpanzees (Pan troglodytes) and human children (Homo sapiens). Anim. Cogn. 8, 151–163 10.1007/s10071-004-0237-8 15490290

[B17] Camerer C. F. (2003). Behavioral Game Theory: Experiments in Strategic Interaction. Princeton, NJ: Princeton University Press

[B18] Carrasco L. Posada S. Colell M. (2009). New evidence on imitation in an enculturated chimpanzee (Pan troglodytes). J. Comp. Psychol. 123, 385–390 10.1037/a0016275 19929107

[B19] Carter R. M. Bowling D. L. Reeck C. Huettel S. A. (2012). A distinct role of the temporal-parietal junction in predicting socially guided decisions. Science 337, 109–111 10.1126/science.1219681 22767930 PMC3563331

[B20] Carter R. M. Huettel S. A. (2013). A nexus model of the temporal–parietal junction. Trends Cogn. Sci. 17, 328–336 10.1016/j.tics.2013.05.007 23790322 PMC3750983

[B192] Caspers S. Zilles K. Laird A. R. Eickhoff S. B. (2010). ALE meta-analysis of action observation and imitation in the human brain. Neuroimage 50, 1148–1167 10.1016/j.neuroimage.2009.12.112 20056149 PMC4981639

[B21] Chang S. W. Gariépy J. F. Platt M. L. (2013). Neuronal reference frames for social decisions in primate frontal cortex. Nat. Neurosci. 16, 243–250 10.1038/nn.3287 23263442 PMC3557617

[B22] Chang S. W. Winecoff A. A. Platt M. L. (2011). Vicarious reinforcement in rhesus macaques (macaca mulatta). Front. Neurosci. 5:27 10.3389/fnins.2011.00027 21516263 PMC3080185

[B23] Chivers D. P. Smith R. J. F. (1994). Fathead minnows, Pimephales promelas, acquire predator recognition when alarm substance is associated with the sight of unfamiliar fish. Anim. Behav. 48, 597–605 10.1006/anbe.1994.1279

[B24] Clayton N. S. Bussey T. J. Dickinson A. (2003). Can animals recall the past and plan for the future? Nat. Rev. Neurosci. 4, 685–691 10.1038/nrn1180 12894243

[B25] Cook M. Mineka S. (1989). Observational conditioning of fear to fear-relevant versus fear-irrelevant stimuli in rhesus monkeys. J. Abnorm. Psychol. 98, 448–459 10.1037/0021-843X.98.4.448 2592680

[B26] Corbetta M. Shulman G. L. (2002). Control of goal-directed and stimulus-driven attention in the brain. Nat. Rev. Neurosci. 3, 201–215 10.1038/nrn755 11994752

[B27] Coussi-Korbel S. Fragaszy D. M. (1995). On the relation between social dynamics and social learning. Anim. Behav. 50, 1441–1453 10.1016/0003-3472(95)80001-8

[B28] Dally J. M. Clayton N. S. Emery N. J. (2008). Social influences on foraging by rooks (Corvus frugilegus). Behaviour 145, 1101–1124 10.1163/156853908784474470

[B29] Deaner R. O. Khera A. V. Platt M. L. (2005). Monkeys pay per view: adaptive valuation of social images by rhesus macaques. Curr. Biol. 15, 543–548 10.1016/j.cub.2005.01.044 15797023

[B30] Dechmann D. K. N. Heucke S. L. Giuggioli L. Safi K. Voigt C. C. Wikelski M. (2009). Experimental evidence for group hunting via eavesdropping in echolocating bats. Proc. Biol. Sci. 276, 2721–2728 10.1098/rspb.2009.0473 19419986 PMC2839959

[B194] di Pellegrino G. Fadiga L. Fogassi L. Gallese V. Rizzolatti G. (1992). Understanding motor events: a neurophysiological study. Exp. Brain Res. 91, 176–180 10.1007/BF00230027 1301372

[B31] Emery N. J. (2000). The eyes have it: the neuroethology, function and evolution of social gaze. Neurosci. Biobehav. Rev. 24, 581–604 10.1016/S0149-7634(00)00025-7 10940436

[B32] Fan J. Gu X. Liu X. Guise K. G. Park Y. Martin L. (2011). Involvement of the anterior cingulate and frontoinsular cortices in rapid processing of salient facial emotional information. Neuroimage 54, 2539–2546 10.1016/j.neuroimage.2010.10.007 20937394 PMC3006498

[B33] Farina W. M. Grüter C. Acosta L. Mc Cabe S. (2007). Honeybees learn floral odors while receiving nectar from foragers within the hive. Naturwissenschaften 94, 55–60 10.1007/s00114-006-0157-3 17021915

[B34] Fiorito G. Scotto P. (1992). Observational Learning in Octopus vulgaris. Science 256, 545–547 10.1126/science.256.5056.545 17787951

[B35] Fogassi L. Ferrari P. F. Gesierich B. Rozzi S. Chersi F. Rizzolatti G. (2005). Parietal lobe: from action organization to intention understanding. Science 308, 662–667 10.1126/science.1106138 15860620

[B37] Freiwald W. A. Tsao D. Y. (2010). Functional compartmentalization and viewpoint generalization within the macaque face-processing system. Science 330, 845–851 10.1126/science.1194908 21051642 PMC3181095

[B38] Frischen A. (2007). Gaze cueing of attention: visual attention, social cognition and individual differences. Psychol. Bull. 133, 694–724 10.1037/0033-2909.133.4.694 17592962 PMC1950440

[B39] Fujii N. Hihara S. Iriki A. (2007). Dynamic social adaptation of motion-related neurons in primate parietal cortex. PLoS ONE 2:e397 10.1371/journal.pone.0000397 17460764 PMC1851098

[B40] Fujii N. Hihara S. Iriki A. (2008). Social cognition in premotor and parietal cortex. Soc. Neurosci. 3, 250–260 10.1080/17470910701434610 18792859

[B187] Fujii N. Hihara S. Nagasaka Y. Iriki A. (2009). Social state representation in prefrontal cortex. Soc. Neurosci. 4, 73–84 10.1080/17470910802046230 18633840

[B41] Funamizu A. Ito M. Doya K. Kanzaki R. Takahashi H. (2012). Uncertainty in action-value estimation affects both action choice and learning rate of the choice behaviors of rats. Eur. J. Neurosci. 35, 1180–1189 10.1111/j.1460-9568.2012.08025.x 22487046 PMC3380560

[B42] Furl N. Hadj-Bouziane F. Liu N. Averbeck B. B. Ungerleider L. G. (2012). Dynamic and static facial expressions decoded from motion-sensitive areas in the macaque monkey. J. Neurosci. 32, 15952–15962 10.1523/JNEUROSCI.1992-12.2012 23136433 PMC3539420

[B43] Galef B. G. Jr. (1995). Why behaviour patterns that animals learn socially are locally adaptive. Anim. Behav. 49, 1325–1334 10.1006/anbe.1995.0164

[B44] Galef B. G. Jr. (1996). Food selection: problems in understanding how we choose foods to eat. Neurosci. Biobehav. Rev. 20, 67–73 10.1016/0149-7634(95)00041-C 8622831

[B45] Galef B. G. Jr. (2003). Social learning of food preferences in rodents: rapid appetitive learning. Curr. Protoc. Neurosci. Chapter 8: Unit 8.5D. 10.1002/0471142301.ns0805ds21 18428585

[B46] Galef B. G. Jr. (2009). Norway rats. Curr. Biol. 19 R884–885 10.1016/j.cub.2009.07.031 19825345

[B47] Galef B. G. Jr. (2012). A case study in behavioral analysis, synthesis and attention to detail: social learning of food preferences. Behav. Brain Res. 231, 266–271 10.1016/j.bbr.2011.07.021 21802448

[B48] Galef B. G. Jr. Beck M. (1985). Aversive and attractive marking of toxic and safe foods by Norway rats. Behav. Neural Biol. 43, 298–310 10.1016/S0163-1047(85)91645-0 3022705

[B49] Galef B. G. Jr. Clark M. M. (1971). Social factors in the poison avoidance and feeding behavior of wild and domesticated rat pups. J. Comp. Physiol. Psychol. 75, 341–357 10.1037/h0030937 5091219

[B50] Galef B. G. Jr. Clark M. M. (1972). Mother's milk and adult presence: two factors determining initial dietary selection by weanling rats. J. Comp. Physiol. Psychol. 78, 220–225 10.1037/h0032293 5061998

[B51] Galef B. G. Jr. Giraldeau L. A. (2001). Social influences on foraging in vertebrates: causal mechanisms and adaptive functions. Anim. Behav. 61, 3–15 10.1006/anbe.2000.1557 11170692

[B52] Galef B. G. Jr. Heiber L. (1976). Role of residual olfactory cues in the determination of feeding site selection and exploration patterns of domestic rats. J. Comp. Physiol. Psychol. 90, 727–739 10.1037/h0077243 987073

[B53] Galef B. G. Jr. Henderson P. W. (1972). Mother's milk: a determinant of the feeding preferences of weaning rat pups. J. Comp. Physiol. Psychol. 78, 213–219 10.1037/h0032186 5061997

[B54] Galef B. G. Jr. Laland K. N. (2005). Social learning in animals: empirical studies and theoretical models. BioScience 55, 489–499 10.1641/0006-3568(2005)055[0489:SLIAES]2.0.CO;2

[B55] Galef B. G. Jr. Marczinski C. A. Murray K. A. Whiskin E. E. (2001). Studies of food stealing by young Norway rats. J. Comp. Psychol. 115, 16–21 10.1037/0735-7036.115.1.16 11334214

[B56] Galef B. G. Jr. Whiskin E. E. (1995). Learning socially to eat more of one food than of another. J. Comp. Psychol. 109, 99–101 10.1037/0735-7036.109.1.99 7705065

[B57] Galef B. G. Jr. Whiskin E. E. (2003). Socially transmitted food preferences can be used to study long-term memory in rats. Learn. Behav. 31, 160–164 10.3758/BF03195978 12882374

[B58] Gerull F. C. Rapee R. M. (2002). Mother knows best: effects of maternal modelling on the acquisition of fear and avoidance behaviour in toddlers. Behav. Res. Ther. 40, 279–287 10.1016/S0005-7967(01)00013-4 11863238

[B184] Gil-da-Costa R. Braun A. Lopes M. Hauser M. D. Carson R. E. Herscovitch P. (2004). Toward an evolutionary perspective on conceptual representation: species-specific calls activate visual and affective processing systems in the macaque. Proc. Natl. Acad. Sci. U.S.A. 101, 17516–17521 10.1073/pnas.0408077101 15583132 PMC536037

[B59] Gläscher J. Büchel C. (2005). Formal learning theory dissociates brain regions with different temporal integration. Neuron 47, 295–306 10.1016/j.neuron.2005.06.008 16039570

[B60] Gordon I. Eilbott J. A. Feldman R. Pelphrey K. A. Vander Wyk B. C. (2013). Social, reward, and attention brain networks are involved when online bids for joint attention are met with congruent versus incongruent responses. Soc. Neurosci. 8, 544–554 10.1080/17470919.2013.832374 24044427

[B61] Gould S. J. Lewontin R. C. (1979). The spandrels of San Marco and the Panglossian paradigm: a critique of the adaptationist programme. Proc. R. Soc. Lond. B Biol. Sci. 205, 581–598 10.1098/rspb.1979.0086 42062

[B62] Göz H. (1942). Über den art-und individualgeruch bei fischen. J. Comp. Physiol. A Neuroethol. Sens. Neural Behav. Physiol. 29, 1–45

[B63] Griffin A. (2004). Social learning about predators: a review and prospectus. Anim. Learn. Behav. 32, 131–140 10.3758/BF03196014 15161148

[B64] Gruber T. Muller M. N. Strimling P. Wrangham R. Zuberbühler K. (2009). Wild chimpanzees rely on cultural knowledge to solve an experimental honey acquisition task. Curr. Biol. 19, 1806–1810 10.1016/j.cub.2009.08.060 19853447

[B65] Gu X. Liu X. Guise K. G. Naidich T. P. Hof P. R. Fan J. (2010). Functional dissociation of the frontoinsular and anterior cingulate cortices in empathy for pain. J. Neurosci. 30, 3739–3744 10.1523/JNEUROSCI.4844-09.2010 20220007 PMC2845539

[B66] Hadj-Bouziane F. Liu N. Bell A. H. Gothard K. M. Luh W. M. Tootell R. B. (2012). Amygdala lesions disrupt modulation of functional MRI activity evoked by facial expression in the monkey inferior temporal cortex. Proc. Natl. Acad. Sci. U.S.A. 109, E3640–E3648 10.1073/pnas.1218406109 23184972 PMC3535608

[B67] Haxby J. V. Hoffman E. A. Gobbini M. I. (2002). Human neural systems for face recognition and social communication. Biol. Psychiatry 51, 59–67 10.1016/S0006-3223(01)01330-0 11801231

[B68] Hayden B. Y. Pearson J. M. Platt M. L. (2009). Fictive reward signals in the anterior cingulate cortex. Science 324, 948–950 10.1126/science.1168488 19443783 PMC3096846

[B195] Hecht E. E. Gutman D. A. Preuss T. M. Sanchez M. M. Parr L. A. Rilling J. K. (2013). Process versus product in social learning: comparative diffusion tensor imaging of neural systems for action execution-observation matching in macaques, chimpanzees, and humans. Cereb. Cortex 23, 1014–1024 10.1093/cercor/bhs097 22539611 PMC3615349

[B183] Heinrich B. (1988). Winter foraging at carcasses by three sympatric corvids, with emphasis on recruitment by the raven, *Corvus corax*. Behav. Ecol. Sociobiol. 23, 141–156 10.1007/BF00300349

[B69] Henrich J. Broesch J. (2011). On the nature of cultural transmission networks: evidence from Fijian villages for adaptive learning biases. Philos. Trans. R. Soc. B Biol. Sci. 366, 1139–1148 10.1098/rstb.2010.0323 21357236 PMC3049092

[B179] Hickok G. (2009). Eight problems for the mirror neuron theory of action understanding in monkeys and humans. J. Cogn. Neurosci. 21, 1229–1243 10.1162/jocn.2009.21189 19199415 PMC2773693

[B70] Hikami K. Hasegawa Y. Matsuzawa T. (1990). Social transmission of food preferences in Japanese monkeys (Macaca fuscata) after mere exposure or aversion training. J. Comp. Psychol. 104, 233–237 10.1037/0735-7036.104.3.233 2225760

[B71] Hooker C. I. Germine L. T. Knight R. T. D'Esposito M. (2006). Amygdala response to facial expressions reflects emotional learning. J. Neurosci. 26, 8915–8922 10.1523/JNEUROSCI.3048-05.2006 16943547 PMC6675340

[B72] Horner V. de Waal F. B. (2009). Controlled studies of chimpanzee cultural transmission. Prog. Brain Res. 178, 3–15 10.1016/S0079-6123(09)17801-9 19874958

[B73] Horner V. Whiten A. (2005). Causal knowledge and imitation/emulation switching in chimpanzees (Pan troglodytes) and children (Homo sapiens). Anim. Cogn. 8, 164–181 10.1007/s10071-004-0239-6 15549502

[B74] Humle T. Matsuzawa T. (2002). Ant-dipping among the chimpanzees of Bossou, Guinea, and some comparisons with other sites. Am. J. Primatol. 58, 133–148 10.1002/ajp.10055 12454957

[B75] Humle T. Snowdon C. T. Matsuzawa T. (2009). Social influences on ant-dipping acquisition in the wild chimpanzees (Pan troglodytes verus) of Bossou, Guinea, West Africa. Anim. Cogn. 12, S37–S48 10.1007/s10071-009-0272-6 19685087

[B76] Iacoboni M. Dapretto M. (2006). The mirror neuron system and the consequences of its dysfunction. Nat. Rev. Neurosci. 7, 942–951 10.1038/nrn2024 17115076

[B77] Iacoboni M. Molnar-Szakacs I. Gallese V. Buccino G. Mazziotta J. C. Rizzolatti G. (2005). Grasping the intentions of others with one's own mirror neuron system. PLoS Biol. 3:e79 10.1371/journal.pbio.0030079 15736981 PMC1044835

[B78] Iwata J. Shima K. Tanji J. Mushiake H. (2013). Neurons in the cingulate motor area signal context-based and outcome-based volitional selection of action. Exp. Brain Res. 229, 407–417 10.1007/s00221-013-3442-3 23455722

[B79] Izuma K. Adolphs R. (2013). Social manipulation of preference in the human brain. Neuron 78, 563–573 10.1016/j.neuron.2013.03.023 23664619 PMC3695714

[B80] Jeon D. Kim S. Chetana M. Jo D. Ruley H. E. Lin S. Y. (2010). Observational fear learning involves affective pain system and Cav1.2 Ca2+ channels in ACC. Nat. Neurosci. 13, 482–488 10.1038/nn.2504 20190743 PMC2958925

[B81] John E. R. Chesler P. Bartlett F. Victor I. (1968). Observation learning in cats. Science 159, 1489–1491 10.1126/science.159.3822.1489 5732493

[B82] Jones J. L. Esber G. R. McDannald M. A. Gruber A. J. Hernandez A. Mirenzi A. (2012). Orbitofrontal cortex supports behavior and learning using inferred but not cached values. Science 338, 953–956 10.1126/science.1227489 23162000 PMC3592380

[B83] Jones R. M. Somerville L. H. Li J. Ruberry E. J. Libby V. Glover G. (2011). Behavioral and neural properties of social reinforcement learning. J. Neurosci. 31, 13039–13045 10.1523/JNEUROSCI.2972-11.2011 21917787 PMC3303166

[B84] Kamphuis S. Dicke P. W. Thier P. (2009). Neuronal substrates of gaze following in monkeys. Eur. J. Neurosci. 29, 1732–1738 10.1111/j.1460-9568.2009.06730.x 19385988

[B85] Kavaliers M. Choleris E. Colwell D. D. (2001). Learning from others to cope with biting flies: social learning of fear-induced conditioned analgesia and active avoidance. Behav. Neurosci. 115, 661–674 10.1037/0735-7044.115.3.661 11439455

[B86] Kawamura S. (1959). The process of sub-culture propagation among Japanese macaques. Primates 2, 43–60 10.1007/BF01666110

[B87] Keller M. Perrin G. Meurisse M. Ferreira G. Lévy F. (2004). Cortical and medial amygdala are both involved in the formation of olfactory offspring memory in sheep. Eur. J. Neurosci. 20, 3433–3441 10.1111/j.1460-9568.2004.03812.x 15610176

[B88] Kennedy D. P. Adolphs R. (2010). Impaired fixation to eyes following amygdala damage arises from abnormal bottom-up attention. Neuropsychologia 48, 3392–3398 10.1016/j.neuropsychologia.2010.06.025 20600184 PMC2949539

[B89] Klein J. T. Deaner R. O. Platt M. L. (2008). Neural correlates of social target value in macaque parietal cortex. Curr. Biol. 18, 419–424 10.1016/j.cub.2008.02.047 18356054 PMC2362498

[B90] Klein J. T. Platt M. L. (2013). Social information signaling by neurons in primate striatum. Curr. Biol. 23, 691–696 10.1016/j.cub.2013.03.022 23562270 PMC3654103

[B91] Klein J. T. Shepherd S. V. Platt M. L. (2009). Social attention and the brain. Curr. Biol. 19, R958–R962 10.1016/j.cub.2009.08.010 19889376 PMC3387539

[B92] Klimecki O. M. Leiberg S. Ricard M. Singer T. (2013). Differential pattern of functional brain plasticity after compassion and empathy training. Soc. Cogn. Affect. Neurosci. [Epub ahead of print]. 10.1093/scan/nst060 23576808 PMC4040103

[B93] Knoch D. Pascual-Leone A. Meyer K. Treyer V. Fehr E. (2006). Diminishing reciprocal fairness by disrupting the right prefrontal cortex. Science 314, 829–832 10.1126/science.1129156 17023614

[B94] Kolling N. Behrens T. E. Mars R. B. Rushworth M. F. (2012). Neural mechanisms of foraging. Science 336, 95–98 10.1126/science.1216930 22491854 PMC3440844

[B95] Koster-Hale J. Saxe R. (2013). Theory of mind: a neural prediction problem. Neuron 79, 836–848 10.1016/j.neuron.2013.08.020 24012000 PMC4041537

[B96] Krebs J. R. Inman A. J. (1992). Learning and foraging – Individuals, groups and populations. Am. Nat. 140, S63–S84 10.1086/285397 19426027

[B97] Krebs J. R. MacRoberts M. H. Cullen J. M. (1972). Flocking and feeding in the great tit parus major – an experimental study. Ibis 114, 507–530 10.1111/j.1474-919X.1972.tb00852.x

[B98] Kumaran D. Melo H. L. Duzel E. (2012). The emergence and representation of knowledge about social and nonsocial hierarchies. Neuron 76, 653–666 10.1016/j.neuron.2012.09.035 23141075 PMC3580285

[B99] Laube I. Kamphuis S. Dicke P. W. Thier P. (2011). Cortical processing of head- and eye-gaze cues guiding joint social attention. Neuroimage 54, 1643–1653 10.1016/j.neuroimage.2010.08.074 20832481

[B100] Leigh R. Oishi K. Hsu J. Lindquist M. Gottesman R. F. Jarso S. (2013). Acute lesions that impair affective empathy. Brain 136, 2539–2549 10.1093/brain/awt177 23824490 PMC3722353

[B101] Lesburguères E. Gobbo O. L. Alaux-Cantin S. Hambucken A. Trifilieff P. Bontempi B. (2011). Early tagging of cortical networks is required for the formation of enduring associative memory. Science 331, 924–928 10.1126/science.1196164 21330548

[B102] Livneh U. Resnik J. Shohat Y. Paz R. (2012). Self-monitoring of social facial expressions in the primate amygdala and cingulate cortex. Proc. Natl. Acad. Sci. U.S.A. 109, 18956–18961 10.1073/pnas.1207662109 23112157 PMC3503171

[B103] Lucchetti C. Lanzilotto M. Perciavalle V. Bon L. (2012). Neuronal activity reflecting progression of trials in the pre-supplementary motor area of macaque monkey: an expression of neuronal flexibility. Neurosci. Lett. 506, 33–38 10.1016/j.neulet.2011.10.043 22040673

[B104] Luncz L. V. Mundry R. Boesch C. (2012). Evidence for cultural differences between neighboring chimpanzee communities. Curr. Biol. 22, 922–926 10.1016/j.cub.2012.03.031 22578420

[B105] Ly M. Haynes M. R. Barter J. W. Weinberger D. R. Zink C. F. (2011). Subjective socioeconomic status predicts human ventral striatal responses to social status information. Curr. Biol. 21, 794–797 10.1016/j.cub.2011.03.050 21530264

[B106] Mars R. B. Sallet J. Schüffelgen U. Jbabdi S. Toni I. Rushworth M. F. (2012). Connectivity-based subdivisions of the human right temporoparietal junction area: evidence for different areas participating in different cortical networks. Cereb. Cortex 22, 1894–1903 10.1093/cercor/bhr268 21955921

[B107] Masi S. Gustafsson E. Saint Jalme M. Narat V. Todd A. Bomsel M. C. (2012). Unusual feeding behavior in wild great apes, a window to understand origins of self-medication in humans: role of sociality and physiology on learning process. Physiol. Behav. 105, 337–349 10.1016/j.physbeh.2011.08.012 21888922

[B108] Masuda A. Aou S. (2009). Social transmission of avoidance behavior under situational change in learned and unlearned rats. PLoS ONE 4:e6794 10.1371/journal.pone.0006794 19710921 PMC2728840

[B109] Mennella J. A. (1995). Mother's milk: a medium for early flavor experiences. J. Hum. Lact. 11, 39–45 10.1177/089033449501100122 7748264

[B110] Menzel E. W. (1974). A group of young chimpanzees in a one-acre field, in Behavior of Non-human Primates: Modern Research Trends, Vol. 5, eds Schrier A. M. Stollnitz F. (New York, NY: Academic Press), 93–153

[B111] Miller E. K. Cohen J. D. (2001). An integrative theory of prefrontal cortex function. Annu. Rev. Neurosci. 24, 167–202 10.1146/annurev.neuro.24.1.167 11283309

[B112] Monfardini E. Gaveau V. Boussaoud D. Hadj-Bouziane F. Meunier M. (2012). Social learning as a way to overcome choice-induced preferences? Insights from humans and rhesus macaques. Front. Neurosci. 6:127 10.3389/fnins.2012.00127 22969703 PMC3432509

[B113] Montague R. (2007). Your Brain is (Almost) Perfect: How we Make Decisions. New York, NY: Plume

[B115] Morgan T. J. Rendell L. E. Ehn M. Hoppitt W. Laland K. N. (2012). The evolutionary basis of human social learning. Proc. Biol. Sci. 279, 653–662 10.1098/rspb.2011.1172 21795267 PMC3248730

[B116] Morishima Y. Schunk D. Bruhin A. Ruff C. C. Fehr E. (2012). Linking brain structure and activation in temporoparietal junction to explain the neurobiology of human altruism. Neuron 75, 73–79 10.1016/j.neuron.2012.05.021 22794262

[B117] Munger S. D. Leinders-Zufall T. McDougall L. M. Cockerham R. E. Schmid A. Wandernoth P. (2010). An olfactory subsystem that detects carbon disulfide and mediates food-related social learning. Curr. Biol. 20, 1438–1444 10.1016/j.cub.2010.06.021 20637621 PMC2929674

[B118] Myowa-Yamakoshi M. Tomonaga M. Tanaka M. Matsuzawa T. (2004). Imitation in neonatal chimpanzees (Pan troglodytes). Dev. Sci. 7, 437–442 10.1111/j.1467-7687.2004.00364.x 15484592

[B180] Newman-Norlund R. D. van Schie H. T. van Zuijlen A. M. Bekkering H. (2007). The mirror neuron system is more active during complementary compared with imitative action. Nat. Neurosci. 10, 817–818 10.1038/nn1911 17529986

[B119] Nicole C. J. Pope S. J. (1999). The effects of demonstrator social status and prior foraging success on social learning in laying hens. Anim. Behav. 57, 163–171 10.1006/anbe.1998.0920 10053083

[B120] Nicolle A. Klein-Flügge M. C. Hunt L. T. Vlaev I. Dolan R. J. Behrens T. E. (2012). An agent independent axis for executed and modeled choice in medial prefrontal cortex. Neuron 75, 1114–1421 10.1016/j.neuron.2012.07.023 22998878 PMC3458212

[B122] Olsson A. Nearing K. I. Phelps E. A. (2007). Learning fears by observing others: the neural systems of social fear transmission. Soc. Cogn. Affect. Neurosci. 2, 3–11 10.1093/scan/nsm005 18985115 PMC2555428

[B123] Olsson A. Phelps E. A. (2007). Social learning of fear. Nat. Neurosci. 10, 1095–1102 10.1038/nn1968 17726475

[B124] Patrick H. Nicklas T. A. (2005). A review of family and social determinants of children's eating patterns and diet quality. J. Am. College Nutr. 24, 83–92 10.1080/07315724.2005.10719448 15798074

[B125] Perry S. (2011). Social traditions and social learning in capuchin monkeys (*Cebus*). Philos. Trans. R. Soc. B 366, 988–996 10.1098/rstb.2010.0317 21357221 PMC3049088

[B126] Perry S. Baker M. Fedigan L. Gros-Louis J. Jack K. MacKinnon K. C. (2003). Social conventions in wild white-faced capuchin monkeys. Curr. Anthropol. 44, 241–268 10.1086/345825

[B127] Pfeiffer M. Nessler B. Douglas R. J. Maass W. (2010). Reward-modulated Hebbian learning of decision making. Neural Comput. 22, 1399–1444 10.1162/neco.2010.03-09-980 20141476

[B128] Rabenold P. P. (1987). Recruitment to food in black vultures: evidence for following from communal roosts. Anim. Behav. 35, 1775–1785 10.1016/S0003-3472(87)80070-2

[B129] Rendell L. Boyd R. Cownden D. Enquist M. Eriksson K. Feldman M. W. (2010). Why copy others? Insights from the social learning strategies tournament. Science 328, 208–213 10.1126/science.1184719 20378813 PMC2989663

[B130] Resulaj A. Kiani R. Wolpert D. M. Shadlen M. N. (2009). Changes of mind in decision-making. Nature 461, 263–266 10.1038/nature08275 19693010 PMC2875179

[B131] Ricciardelli P. Bricolo E. Aglioti S. M. Chelazzi L. (2002). My eyes want to look where your eyes are looking: exploring the tendency to imitate another individual's gaze. Neuroreport 13, 2259–2264 10.1097/00001756-200212030-00018 12488807

[B132] Ross R. S. Eichenbaum H. (2006). Dynamics of hippocampal and cortical activation during consolidation of a nonspatial memory. J. Neurosci. 26, 4852–4859 10.1523/JNEUROSCI.0659-06.2006 16672659 PMC6674163

[B185] Ross R. S. McGaughy J. Eichenbaum H. (2005). Acetylcholine in the orbitofrontal cortex is necessary for the acquisition of a socially transmitted food preference. Learn. Memory 12, 302–306 10.1101/lm.91605 15897258 PMC1142459

[B133] Roy A. Shepherd S. V. Platt M. L. (2012). Reversible inactivation of pSTS suppresses social gaze following in the macaque (Macaca mulatta). Soc. Cogn. Affect. Neurosci. 9, 209–217 10.1093/scan/nss123 23171617 PMC3907927

[B134] Rozzi S. Ferrari P. F. Bonini L. Rizzolatti G. Fogassi L. (2008). Functional organization of inferior parietal lobule convexity in the macaque monkey: electrophysiological characterization of motor, sensory and mirror responses and their correlation with cytoarchitectonic areas. Eur. J. Neurosci. 28, 1569–1588 10.1111/j.1460-9568.2008.06395.x 18691325

[B135] Rushworth M. F. Hadland K. A. Gaffan D. Passingham R. E. (2003). The effect of cingulate cortex lesions on task switching and working memory. J. Cogn. Neurosci. 15, 338–353 10.1162/089892903321593072 12729487

[B186] Sallet J. Mars R. B. Noonan M. P. Andersson J. L. O'Reilly J. X. Jbabdi S. (2011). Social network size affects neural circuits in macaques. Science 334, 697–700 10.1126/science.1210027 22053054

[B136] Samson D. Apperly I. A. Chiavarino C. Humphreys G. W. (2004). Left temporoparietal junction is necessary for representing someone else's belief. Nat. Neurosci. 7, 499–500 10.1038/nn1223 15077111

[B137] Saxe R. Kanwisher N. (2003). People thinking about thinking people. The role of the temporo-parietal junction in theory of mind. Neuroimage 19, 1835–1842 10.1016/S1053-8119(03)00230-1 12948738

[B138] Schilbach L. Eickhoff S. B. Cieslik E. Shah N. J. Fink G. R. Vogeley K. (2011). Eyes on me: an fMRI study of the effects of social gaze on action control. Soc. Cogn. Affect. Neurosci. 6, 393–403 10.1093/scan/nsq067 20705602 PMC3150858

[B139] Schultz W. (1998). Predictive reward signal of dopamine neurons. J. Neurophysiol. 80, 1–27 9658025 10.1152/jn.1998.80.1.1

[B140] Schultz W. Dayan P. Montague R. R. (1997). A neural substrate of prediction and reward. Science 275, 1583–1599 10.1126/science.275.5306.1593 9054347

[B141] Seo H. Lee D. (2008). Cortical mechanisms for reinforcement learning in competitive games. Philos. Trans. R. Soc. Lond. B Biol. Sci. 363, 3845–3857 10.1098/rstb.2008.0158 18829430 PMC2607365

[B142] Shepherd S. V. Klein J. T. Deaner R. O. Platt M. L. (2009). Mirroring of attention by neurons in macaque parietal cortex. Proc. Natl. Acad. Sci. U.S.A. 106, 9489–9494 10.1073/pnas.0900419106 19470477 PMC2685741

[B143] Sherry D. F. Galef B. G. Jr. (1984). Cultural transmission without imitation: milk bottle opening by birds. Anim. Behav. 32, 937–938 10.1016/S0003-3472(84)80185-2

[B144] Sherwin C. M. Heyes C. M. Nicol C. J. (2002). Social learning influences the preferences of domestic hens for novel food. Anim. Behav. 63, 933–942 10.1006/anbe.2002.2000

[B145] Shima K. Isoda M. Mushiake H. Tanji J. (2007). Categorization of behavioural sequences in the prefrontal cortex. Nature 445, 315–318 10.1038/nature05470 17183266

[B146] Shima K. Tanji J. (1998). Both supplementary and presupplementary motor areas are crucial for the temporal organization of multiple movements. J. Neurophysiol. 80, 3247–3260 9862919 10.1152/jn.1998.80.6.3247

[B147] Shima K. Tanji J. (2006). Binary-coded monitoring of a behavioral sequence by cells in the pre-supplementary motor area. J. Neurosci. 26, 2579–2582 10.1523/JNEUROSCI.4161-05.2006 16510736 PMC6793666

[B148] Singer T. Seymour B. O'Doherty J. Kaube H. Dolan R. J. Frith C. D. (2004). Empathy for pain involves the affective but not sensory components of pain. Science 303, 1157–1162 10.1126/science.1093535 14976305

[B149] Slocombe K. E. Zuberbühler K. (2006). Food-associated calls in chimpanzees: responses to food types or food preferences? Anim. Behav. 72, 989–999 10.1016/j.anbehav.2006.01.030

[B193] Sowden S. Catmur C. (2013). The role of the right temporoparietal junction in the control of imitation. Cereb. Cortex. [Epub ahead of print]. 10.1093/cercor/bht306 24177989 PMC4380005

[B150] Spence K. W. (1937). Experimental studies of learning and the higher mental processes in infra-human primates. Psychol. Bull. 34, 806–850 10.1037/h0061498

[B181] Stanley D. A. Adolphs R. (2013). Toward a neural basis for social behavior. Neuron 80, 816–826 10.1016/j.neuron.2013.10.038 24183030 PMC3940487

[B151] Steinberg E. E. Keiflin R. Boivin J. R. Witten I. B. Deisseroth K. Janak P. H. (2013). A causal link between prediction errors, dopamine neurons and learning. Nat. Neurosci. 16, 966–973 10.1038/nn.3413 23708143 PMC3705924

[B152] Stoeger A. S. Mietchen D. Oh S. de Silva S. Herbst C. T. Kwon S. (2012). An Asian elephant imitates human speech. Curr. Biol. 22, 2144–2148 10.1016/j.cub.2012.09.022 23122846 PMC3548412

[B153] Stolk A. Verhagen L. Schoffelen J. M. Oostenveld R. Blokpoel M. Hagoort P. (2013). Neural mechanisms of communicative innovation. Proc. Natl. Acad. Sci. U.S.A. 110, 14574–14579 10.1073/pnas.1303170110 23959895 PMC3767563

[B154] Subiaul F. Cantlon J. F. Holloway R. L. Terrace H. S. (2004). Cognitive imitation in rhesus macaques. Science 305, 407–410 10.1126/science.1099136 15256673

[B155] Suzuki S. Harasawa N. Ueno K. Gardner J. L. Ichinohe N. Haruno M. (2012). Learning to simulate others' decisions. Neuron 74, 1125–1137 10.1016/j.neuron.2012.04.030 22726841

[B156] Swaney W. Kendal J. Capon H. Brown C. Laland K. N. (2001). Familiarity facilitates social learning of foraging behaviour in the guppy. Anim. Behav. 62, 591–598 10.1006/anbe.2001.1788

[B157] Tanji J. Hoshi E. (2008). Role of the lateral prefrontal cortex in executive behavioral control. Physiol. Rev. 88, 37–57 10.1152/physrev.00014.2007 18195082

[B158] Tazumi T. Hori E. Maior R. S. Ono T. Nishijo H. (2010). Neural correlates to seen gaze-direction and head orientation in the macaque monkey amygdala. Neuroscience 169, 287–301 10.1016/j.neuroscience.2010.04.028 20412835

[B159] Tennie C. Call J. Tomasello M. (2012). Untrained chimpanzees (Pan troglodytes schweinfurthii) fail to imitate novel actions. PLoS ONE 7:e41548 10.1371/journal.pone.0041548 22905102 PMC3414512

[B160] Thornton A. (2008). Social learning about novel foods in young meerkats. Anim. Behav. 76, 1411–1421 10.1016/j.anbehav.2008.07.007

[B189] Tomasello M. Savage-Rumbaugh S. Kruger A. C. (1993). Imitative learning of actions on objects by children, chimpanzees, and enculturated chimpanzees. Child Dev. 64, 1688–1705 10.1111/j.1467-8624.1993.tb04207.x 8112113

[B161] Tsao D. Y. Moeller S. Freiwald W. A. (2008). Comparing face patch systems in macaques and humans. Proc. Natl. Acad. Sci. U.S.A. 105, 19514–19519 10.1073/pnas.0809662105 19033466 PMC2614792

[B162] van den Bos R. Jolles J. W. Homberg J. R. (2013). Social modulation of decision-making: a cross-species review. Front. Hum. Neurosci. 7:301 10.3389/fnhum.2013.00301 23805092 PMC3693511

[B163] van den Stock J. Vandenbulcke M. Sinke C. B. Goebel R. de Gelder B. (2013). How affective information from faces and scenes interacts in the brain. Soc. Cogn. Affect. Neurosci. [Epub ahead of print]. 10.1093/scan/nst138 23956081 PMC4187263

[B164] van de Waal E. Borgeaud C. Whiten A. (2013). Potent social learning and conformity shape a wild primate's foraging decisions. Science 340, 483–485 10.1126/science.1232769 23620053

[B165] van Schaik C. P. Burkart J. M. (2011). Social learning and evolution: the cultural intelligence hypothesis. Philos. Trans. R. Soc. Lond. B Biol. Sci. 366, 1008–1016 10.1098/rstb.2010.0304 21357223 PMC3049085

[B166] Venkatraman V. Rosati A. G. Taren A. A. Huettel S. A. (2009). Resolving response, decision, and strategic control: evidence for a functional topography in dorsomedial prefrontal cortex. J. Neurosci. 29, 13158–13164 10.1523/JNEUROSCI.2708-09.2009 19846703 PMC2801415

[B188] Wang F. Zhu J. Zhu H. Zhang Q. Lin Z. Hu H. (2011). Bidirectional control of social hierarchy by synaptic efficacy in medial prefrontal cortex. Science 334, 693–697 10.1126/science.1209951 21960531

[B167] Watson K. K. Platt M. L. (2012). Social signals in primate orbitofrontal cortex. Curr. Biol. 22, 2268–2273 10.1016/j.cub.2012.10.016 23122847 PMC3518589

[B168] Whiten A. (1998). Imitation of the sequential structure of actions by chimpanzees (Pan troglodytes). J. Comp. Psychol. 112, 270–281 10.1037/0735-7036.112.3.270 9770315

[B169] Whiten A. (2005). The second inheritance system of chimpanzees and humans. Nature 437, 52–55 10.1038/nature04023 16136127

[B170] Whiten A. Custance D. M. Gömez J. C. Teixidor P. Bard K. A. (1996). Imitative learning of artificial fruit processing in children (*Homo sapiens*) and chimpanzees (*Pan troglodytes*). J. Comp. Psychol. 110, 3–14 10.1037/0735-7036.110.1.3 8851548

[B171] Wisdom T. N. Song X. Goldstone R. L. (2013). Social learning strategies in networked groups. Cogn. Sci. 37, 1383–1425 10.1111/cogs.12052 23845020

[B172] Wood D. (1989). Social interaction as tutoring, in Interaction in Human Development, ed Bornstein M. Bruner Erlbaum J. (London: Basil Blackwell Ltd.), 59–80

[B173] Yorzinski J. L. Platt M. L. (2010). Same-sex gaze attraction influences mate-choice copying in humans. PLoS ONE 5:e9115 10.1371/journal.pone.0009115 20161739 PMC2817731

[B174] Yoshida K. Saito N. Iriki A. Isoda M. (2011). Representation of others' action by neurons in monkey medial frontal cortex. Curr. Biol. 21, 249–253 10.1016/j.cub.2011.01.004 21256015

[B175] Yoshida K. Saito N. Iriki A. Isoda M. (2012). Social error monitoring in macaque frontal cortex. Nat. Neurosci. 15, 1307–1312 10.1038/nn.3180 22864610

[B176] Young M. E. Mizzau M. Mai N. T. Sirisegaram A. Wilson M. (2009). Food for thought. What you eat depends on your sex and eating companions. Appetite 53, 268–271 10.1016/j.appet.2009.07.021 19646494

[B177] Zentall T. R. (2012). Perspectives on observational learning in animals. J. Comp. Psychol. 126, 114–128 10.1037/a0025381 21895354

[B178] Zink C. F. Tong Y. Chen Q. Bassett D. S. Stein J. L. Meyer-Lindenberg A. (2008). Know your place: neural processing of social hierarchy in humans. Neuron 58, 273–283 10.1016/j.neuron.2008.01.025 18439411 PMC2430590

